# Eliciting brain waves of people with cognitive impairment during meditation exercises using portable electroencephalography in a smart-home environment: a pilot study

**DOI:** 10.3389/fnagi.2023.1167410

**Published:** 2023-05-30

**Authors:** Ioulietta Lazarou, Vangelis P. Oikonomou, Lampros Mpaltadoros, Margarita Grammatikopoulou, Vasilis Alepopoulos, Thanos G. Stavropoulos, Anastasios Bezerianos, Spiros Nikolopoulos, Ioannis Kompatsiaris, Magda Tsolaki, RADAR-AD Consortium

**Affiliations:** ^1^Centre for Research and Technology Hellas (CERTH), Information Technologies Institute (ITI), Thessaloniki, Greece; ^2^1st Department of Neurology, Faculty of Health Sciences, G.H. “AHEPA”, School of Medicine, Aristotle University of Thessaloniki (AUTH), Thessaloniki, Greece; ^3^Greek Association of Alzheimer’s Disease and Related Disorders (GAADRD), Thessaloniki, Greece; ^4^Laboratory of Neurodegenerative Diseases, Center for Interdisciplinary Research and Innovation (CIRI–AUTh), Aristotle University of Thessaloniki, Thessaloniki, Greece

**Keywords:** meditation, Alzheimer’s disease, mindfulness, Kirtan Kriya, electroencephalography, subjective cognitive decline, mild cognitive impairment, smart home

## Abstract

**Objectives:**

Meditation imparts relaxation and constitutes an important non-pharmacological intervention for people with cognitive impairment. Moreover, EEG has been widely used as a tool for detecting brain changes even at the early stages of Alzheimer’s Disease (AD). The current study investigates the effect of meditation practices on the human brain across the AD spectrum by using a novel portable EEG headband in a smart-home environment.

**Methods:**

Forty (40) people (13 Healthy Controls—HC, 14 with Subjective Cognitive Decline—SCD and 13 with Mild Cognitive Impairment—MCI) participated practicing Mindfulness Based Stress Reduction (Session 2-MBSR) and a novel adaptation of the Kirtan Kriya meditation to the Greek culture setting (Session 3-KK), while a Resting State (RS) condition was undertaken at baseline and follow-up (Session 1—RS Baseline and Session 4—RS Follow-Up). The signals were recorded by using the Muse EEG device and brain waves were computed (alpha, theta, gamma, and beta).

**Results:**

Analysis was conducted on four-electrodes (AF7, AF8, TP9, and TP10). Statistical analysis included the Kruskal–Wallis (KW) nonparametric analysis of variance. The results revealed that both states of MBSR and KK lead to a marked difference in the brain’s activation patterns across people at different cognitive states. Wilcoxon Signed-ranks test indicated for HC that theta waves at TP9, TP10 and AF7, AF8 in Session 3-KK were statistically significantly reduced compared to Session 1-RS *Z* = –2.271, *p* = 0.023, *Z* = −3.110, *p* = 0.002 and *Z* = −2.341, *p* = 0.019, *Z* = −2.132, *p* = 0.033, respectively.

**Conclusion:**

The results showed the potential of the parameters used between the various groups (HC, SCD, and MCI) as well as between the two meditation sessions (MBSR and KK) in discriminating early cognitive decline and brain alterations in a smart-home environment without medical support.

## 1. Introduction

More than 55 million people worldwide are affected with Alzheimer’s disease (AD), which is the most common neurodegenerative disease, while according to the WHO, this number is anticipated to reach 139 million cases by 2050 (S. Report, 2021). However, the cause of AD and its preclinical stages, such as Mild Cognitive Impairment (MCI) and Subjective Cognitive Decline (SCD), are still unclear, and no effective treatment has yet been suggested (Petersen et al., 2001; Albert et al., 2011; Stewart, 2012; Bessi et al, 2018; Yue et al., 2021), although early detection of these conditions is of great scientific interest. Every year, 10–15 percent of MCI patients develop AD, and more than half of all converts are expected to develop AD within 5 years (Gauthier et al., 2006; Tarnanas et al., 2015). However, a number of non-pharmacological approaches have also been suggested since they have lower dangers and side effects. On the other hand, it is still a top scientific priority to identify cognitive deterioration as soon as possible to forestall progress of cognitive dysfunction and AD. Thus, electroencephalography (EEG), which has been extensively investigated for its advantages in identifying early cognitive decline, appears to be a potential approach in this regard as offering a non-invasive and simple instrument for early detection of brain activity abnormalities across the AD spectrum (Lazarou et al., 2019a, 2020). Electroencephalogram (EEG) has been used as a tool for diagnosing AD and several techniques have been adopted in order to detect EEG abnormalities in AD patients. In this direction, taking into consideration previous studies have shed light to the clinical importance of the EEG by exploring brain frequencies, ERPs or more advanced metrics of brain connectome based on graph theory (Lazarou et al. 2019b, 2020) which can improve our understanding on the complex organization of the human brain at the early stages of cognitive decline. Regarding brain waves, EEG related studies have shown a power increase of delta and theta power in cognitively impaired people and a parallel power decrease in alpha and beta activity compared with those of normal elderly subjects during resting state activity (Aftanas and Golocheikine, 2001; Lal and Craig, 2002 Aftanas and Golocheikine, 2003; Lutz et al., 2008; Foxe and Snyder, 2011; Wells et al., 2013; Snyder et al., 2015; Tsoneva et al., 2015; Deolindo et al., 2020; Bentley et al., 2022; Lazarou et al., 2022). Recent scientific data suggest that specific EEG markers are correlated to the prognosis of conversion. Such markers are the increased theta/gamma ratio, the reduction of alpha frequency which seems to be associated with conversion to AD. In addition, in MCI and AD subjects resting state posterior delta and alpha EEG rhythms seem to be more sensitive to AD neurodegenerative processes (Osterrieth, 1944). Babiloni et al. in their work suggested the hypothesis that, in MCI and AD subjects, abnormalities of EEG rhythms exist due to the cortical atrophy across the disease. Their results showed that abnormalities of resting state cortical EEG rhythms are strictly related to neurodegeneration and cognition (Babiloni et al., 2013).

Utilizing meditation techniques is one type of non-pharmacological intervention for cognitive impairment caused by AD. In complementary and alternative medicine, meditation is a mind–body approach that focuses on sustaining one’s attention in order to promote mental improvement, concentration, inner calm, and pleasant emotions (Pandey and Miyapuram, 2021). As a result, meditation is considered as a cognitively stimulating exercise and a potential AD prevention technique. Popular methods of meditation include transcendental meditation, Zen meditation, mindfulness-based stress reduction (MBSR), and Kirtan Kriya (KK) (Anwar et al., 2018). Additionally, these methods involve either general relaxation or enhancement of focused attention. Studies looking at how meditation affects cognitive performance have employed morphometric neuroimaging methods, such MRI, to look at possible structural changes in the brain. Many have investigated aspects of functional connectivity in the brain using EEG, event-related potentials (ERPs), positron emission tomography (PET), and functional fMRI (Yoshida et al., 2020). However, this research remains in its infancy.

Researchers and medical experts are attempting to ascertain whether there is a relationship between meditation practice and early cognitive impairment due to the possible curative effects that meditation may have on the mind and body. In order to generate good thoughts, unwind the mind, relieve tension, or improve the mind–body connection, one must learn to manage their mind through the practice of meditation. For instance, MBSR is a type of meditation that emphasizes accepting one’s sensations and thoughts while quietly noticing one’s consciousness in the present moment (Tang et al., 2015; Kora et al., 2021). In Vago and Silbersweig’s model (Vago and David, 2012), meta self-awareness, self-regulation, and self-transcendence are three main self-related capacities that are developed through MBSR practice. It entails a process of dealing with painful or dysphoric events with more dispassion and less reactivity as a professional intervention (Lomas et al., 2015). Additionally, it has been suggested that MBSR meditation encourages the growth of present-moment consciousness in a kind, non-judgmental manner. While many people find the benefits of meditation appealing, neuroscientists have been investigating the impact of MBSR on the human brain, and their findings are shedding light on new techniques that might improve cognitive function. There is evidence that meditation can alter the physical structure of the human brain (Lomas et al., 2015; Tang et al., 2015; Dwivedi et al., 2021). Using MRI technology, it was discovered that the hippocampus, which is primarily engaged in cognitive processes of learning and memory, spatial orientation, and emotion regulation, experiences an increase in brain cell volume after MBSR. Also, the temporo-parietal area, which is involved in feelings of empathy and compassion, also showed increases, but the amygdala, which is involved in feelings of fear, anxiety, and stress, showed decrease in brain cell volume. Those changes show that meditation not only alters the brain but also the cognitive function and behavior, as shown, corresponded to the participants’ self-reports of their stress levels (Lazar et al., 2005). The majority of the MCI were not only able to learn mindfulness meditation but also had higher levels of acceptance, self-efficacy, social engagement, and lower levels of chronic stress (Innes et al., 2021; Khalsa and Newberg, 2021). In recent MBSR studies, it was shown that patients and caregivers both reported higher quality of life ratings, fewer depressive symptoms, and better subjective sleep quality (Lensen et al., 2021; Maddock and Blair, 2021).

On the other hand, KK constitutes an active form of meditation and can relax a person through a number of different processes. By elevating significant neurotransmitters, practice may help regulate brain synapses (i.e., acetylcholine, norepinephrine, etc.; Innes et al., 2018, 2021; Khalsa and Newberg, 2021). This is important since prior research contends that synaptic dysfunction is a distinctive feature of AD. Specific brain regions are stimulated when the practitioner uses their fingertips in combination with sound, as shown on Single-Photon Emission Computed Tomography (SPECT) scans. Increased blood flow from such brain stimulation could have positive effects against neurodegeneration. In the first study that looked at the effects of KK in people with cognitive impairment (SCD, MCI, and AD), it was discovered that KK was beneficial on cognitive performance and increase cerebral blood flow (Innes et al., 2016, 2017). As a result, meditation may be a useful strategy in the fight against and prevention of AD. The majority of research on the effects of meditation, however, has been conducted over an extended period of time and in carefully monitored clinical experimental settings. There is currently no cross-sectional study that has investigated the advantages of remote meditation practices in a home-based setting.

On the other hand, an electrophysiological tool with significant potential for accurately identifying aged individuals across the AD spectrum while performing passive as well as active behaviors like meditation, is EEG which constitutes a non-invasive brain imaging technique that analyzes spatiotemporal features of underlying brain activity. Theta and alpha wave associated synchronization has been a frequently observed aspect of meditation (Kaur and Singh, 2015), which are regarded as markers of internally-directed attention processing (Ahani et al., 2014). Such synchronization has been observed across different meditation practices, including MBSR, as well as practices such as transcendental meditation. However, different types of meditation practices have been associated with particular brain waves activation patterns, reflecting the form of attention as a cognitive function (Cahn and Polich, 2006). Additionally, EEG spectral features are a useful tool for examining the activity of the brain, and it has been demonstrated that, generally, greater activity occurs in the alpha and theta bands during the practice of meditation (Baijal and Srinivasan, 2010). Spectral features are also used to discriminate between meditation and control conditions (Aftanas and Golocheikine, 2002; Xue et al., 2014). For example, MBSR has been associated with increased alpha power, while focused attention has been associated with increased gamma activity, whereas idiosyncratic meditation with decreased alpha and beta (Cahn and Polich, 2006; Kaur and Singh, 2015; Sharma et al., 2019). Last but not least in recent reviews (Lomas et al., 2015; Lee et al., 2018), the authors stressed the importance of future meditative and EEG studies scrutinizing the features of the meditative state instead of analyzing it as an intervention.

The above mentioned and more electrophysiological studies across AD spectrum use multichannel EEG devices. Traditional EEG recording methods are relatively time-consuming, require extensive training, and the recording systems are cumbersome. Moreover, the widely used wet-gel electrodes in clinical EEG applications typically require some kind of skin preparation. In addition, when considering older populations and people dealing with conditions such as AD, stroke etc., lack of cooperation can prolong the already time-consuming procedure of electrode placement. Hence, these medical EEG systems are not optimized for use with people with dementia in daily, at home, practice. Due to these, mobile, rapid and cost effective technologies are emerging as valuable tools for assessing various conditions and with the capability to be used at home. Therefore, small and wireless EEG systems, with dry electrodes and limited channels have become popular and have been used in a number of studies, as they are portable, low cost and easy to use.

Α number of studies combine the portable Muse EEG^1^ with assessing mindfulness and exploring brain activity. In a recent study (Hunkin et al., 2021), a series of meditation exercises were implemented to explore the feasibility of the device to support mindfulness assessment. Another study compared three different meditation techniques in order to evaluate their effectiveness (Sharma et al., 2022). Also, mindfulness training with Muse was associated, through self-reported information and EEG derived indicators, with improvements in Obsessive Compulsive disorder (OCD) symptoms (Hawley et al., 2021). In particular, a recent study explored meditation across 53 novice meditators for 1 month, including Muse EEG. Following the 1-month, both app and muse groups showed significantly reduced distress and increased mindfulness scores following the intervention (Acabchuk et al., 2021). The Muse EEG device is a promising tool that allows the collection of data in a non-clinical environment and without the supervision of a health professional (Acabchuk et al., 2021; Kora et al., 2021). In combination with mindfulness approaches, the collected EEG data could be utilized as valuable indicators to explore brain activity in real environments.

Considering the importance of the dynamics of brain waves during different meditative states in elderly population across AD spectrum, the current study aimed to address for the first time whether different meditation sessions (MBSR, KK) affect brain waves in preclinical stages of AD (MCI and SCD) compared to Healthy Controls (HC) by using a portable EEG (Muse) in a smart home environment. We also hypothesized that meditation may also benefit the more severely affected posterior areas of the brain of participants with SCD and MCI. As part of the Tier 3 study of RADAR-AD project^2^ was to monitor remotely participants across different cognitive states while performing tailored ADL activities and perform meditation with remote monitoring technologies. In particular, we examined the brain waves of HC, SCD and MCI with respect to four meditation conditions: resting state before and after the meditation sessions (Session 1-RS Baseline, Session 2-MBSR, Session 3-KK and Session 4-RS Follow-Up). In detail, the aims of the present pilot study were:

- To investigate and differentiate for the first time different meditations and EEG signals of people across the preclinical stages of AD spectrum (MCI, SCI) by using a low cost portable EEG device.
- To explore brain activation patterns acquired in a smart-home environment with respect to meditation practices.
- To verify whether MBSR and KK constitute a beneficial intervention for preclinical stages of the AD spectrum, given that meditation techniques activate particular brain areas and thus could have potential effect on reversing cognitive decline.

In the present pilot study, we followed the Patient intervention comparator outcome (PICO) process (Eriksen and Frandsen, 2018). We hypothesized that MBSR and KK could be indicative marks of distinguishing preclinical stages of MCI and SCD based on their neuro-responses while performing the meditation exercises and resting state at baseline and follow-up (Table 1).

**Table 1:** PICO pilot study design.

Participants	Intervention	Comparator	Outcome
Healthy Controls Subjective Cognitive Decline Mild Cognitive Impairment	Portable EEG Muse at Smart Home MBSR KK	Resting state at baseline and follow-up	alpha, beta and theta waves between groups Comparison between different meditation states

## 2. Materials and methods

### 2.1. Participants

**Figure 1:**
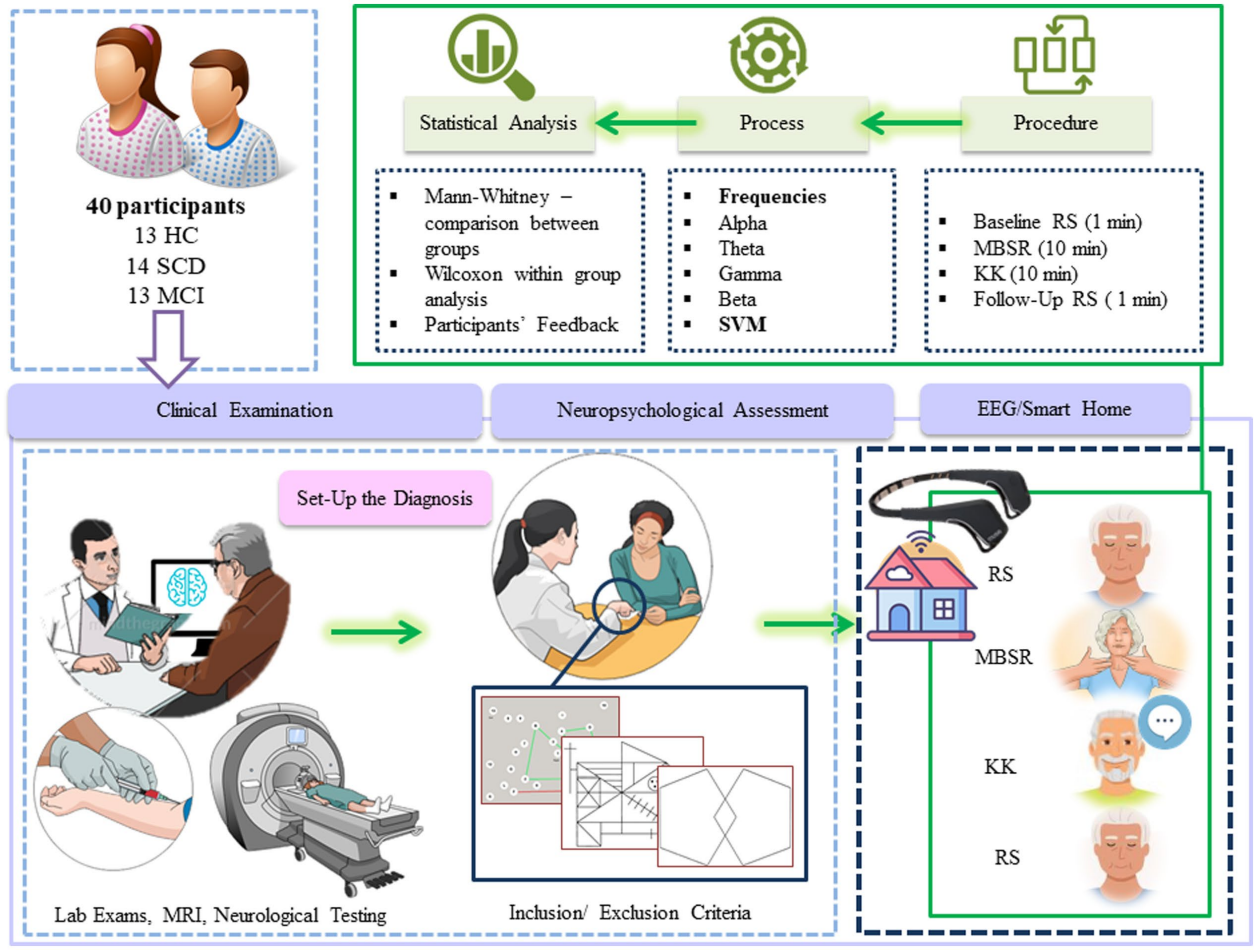
Study pipeline.

In total, 40 participants were recruited from the Greek Association of Alzheimer’s Disease and Related Disorders (GAADRD)^3^ and wide community audience. The study, carried out in accordance with the Declaration of Helsinki, had received approval by the Ethical committee of CERTH (ETH.COM 54/17-06-2020) and Scientific and Ethics Committee of GAADRD (242/2022 ΑΙ_07/10/2021), while a written informed consent was obtained from all participants prior to their participation in the study. The diagnosis of HC, SCD and MCI was set by a neuropsychiatrist, specialized in dementia, according to the structural magnetic resonance imaging (MRI), medical history, neuropsychological tests and neurological examination (Figure 1).

In detail, the HC group consisted of 13 participants, SCD group of 14 and MCI of 13, having similar range of age and education. We have used the power sample calculation formula suggested by (S. Report, 2021) and the number of 40 participants for the present pilot study seems adequate. The total duration of the study was approximately 3 months. Details, regarding the age distribution among the groups are presented in Table 2. The MCI group fulfilled the Petersen criteria (Petersen et al., 2009), while the SCD group met IWG-2 Guidelines (Dubois et al., 2014) as well as the SCD-I Working Group instructions (Molinuevo et al., 2017). Regarding the SCD, we excluded participants with confounding factors based on blood tests (hormonal disorders, vitamins deficiency, etc.) and structural MRI for SCD and MCI (vascular/demyelinating lesions, tumors, anatomical variations etc.). All of the above were taken under consideration for the recruitment process, since they could affect our sample performance and signal elicitation. Additional inclusion criteria for the SCD and HC participants included having a normal general medical, neurological and neuropsychological examination. Exclusion criteria included left-handedness and severe psychiatric, physical or other neurological disorder, illness or any other somatic disorder, which may cause cognitive impairment.

**Table 2:** Mean ± SD (standard deviation) and *p* value of one-way ANOVA of demographic characteristics and neuropsychological assessment among participants (HC = 13, SCD = 14, MCI = 13).

	HC	SCD	MCI	*p* Value
Demographic characteristics				
Age	64.23 (6.26)	65.43 (7.32)	72,08 (8.12)	0.734
Gender (M:F)	2:11	4:10	4:9	0.522
Years of education	14.15 (2.76)	14.57 (1.99)	11.69 (3.54)	0.253
Neuropsychological tests				
MMSE	29.31^**^	27.86^***^	26.08	**0.0001**
MoCA	26.46^**^	25.43^***^	19.92	**0.0001**
FRSSD total score	2.23^*^	2.64^***^	3.92	**0.0331**
FUCAS total score	42.00^**^	42.29^***^	44.23	**0.0001**
ROCFT—copy	34.769^**^	32.929	30.808	**0.0031**
ROCFT—delayed recall	17.577^**^	19.536^***^	11.038	**0.0041**
RBMT-immediate recall	15.231^**^	13.571^***^	10.923	**0.0061**
RBMT-delayed recall	13.808^**^	11.750^***^	7.462	**0.0001**
RAVLT-total score	44.54^*^	38.57	33.38	**0.0211**
TMT-part B	149.23^**^	154.50	224.54	**0.0161**
FAS	11.2754^**^	10.0893	8.6469	**0.0041**
ADAS-Cog	9.6077^**^	12.0000^***^	18.6223	0.8441

* HC vs MCI, *p* < 0.05.

** HC vs MCI, *p* < 0.01.

*** SCD vs MCI, *p* < 0.05.

*p*-values under 0.05 are displayed in bold.

Appropriate informed consent from all participants was obtained prior to their inclusion into the study. Participant Information Forms were prepared according to ICH-GCP requirements [European Medicines Agency (EMA), 2016] and data protection regulations. The information was presented in a manner to enable people to fully understand the relevant aspects of the study. All potential participants were informed about the relevance and the content of the study as well as about the protection of their personal rights, data management and privacy. Moreover, the study was conducted during the pandemic (2021); therefore solely participants that were fully vaccinated were included (validated vaccination certificates with verified app) while after each participant’s visit decontamination by experts took place at the smart home to ensure the safety of the participants.

### 2.2. Clinical and neuropsychological assessment

All participants underwent physical and neurological examination (MRI and blood exams to set the diagnosis) as well as a thorough neuropsychological evaluation prior to their participation with a standardized neuropsychological battery, an insightful psychological interview and medical history using the Structured Clinical Interview for DSM-V Axis I Disorders Clinical Version (SCID-CV) (First et al., 2004). In particular, the following neuropsychological battery was implemented in order to assess cognitive status comprehensively and evaluate aspects like working memory, executive functioning, attention, memory and language: (a) the Greek version of Mini Mental State Examination (MMSE) (Folstein et al., 1975), (b) Functional Rating Scale for Dementia (FRSSD; Hutton, 1987), (c) Functional and Cognitive Assessment Test (FUCAS; Kounti et al., 2006), (d) Trail Making Test part-B (Tombaugh, 2004), (e) RBMT-story Direct and delayed recall (Wilson et al., 1989), (f) Rey Osterrieth Complex Figure Test copy and delay recall (ROCFT-copy and delayed recall; Osterrieth, 1944), (g) Rey Auditory Verbal Learning Test (RAVLT), and (h)Verbal Fluency test FAS Evaluation of mood and behavior took place both from the interview data and the participant and caregiver answers to the relative brief self-report tools, the Neuropsychiatric Inventory (NPI; Cummings et al., 1994) and the Perceived Stress Scale (PSS; Cohen et al., 1983). Notably, in each of the neuropsychological tests the cognitive performance of the three groups was assessed (Table 2). In Table 2, mean, standard deviation and *p* value of one-way ANOVA of demographic characteristics and neuropsychological assessment among participants (HC = 13, SCD = 14, MCI = 13) are presented. Superscripts indicate whether the difference between groups (HC vs. SCD, HC vs. MCI and SCD vs. MCI) is statistical significant using the independent sample *t*-test.

### 2.3. Smart-home CERTH/ITI set-up and settings

**Figure 2:**
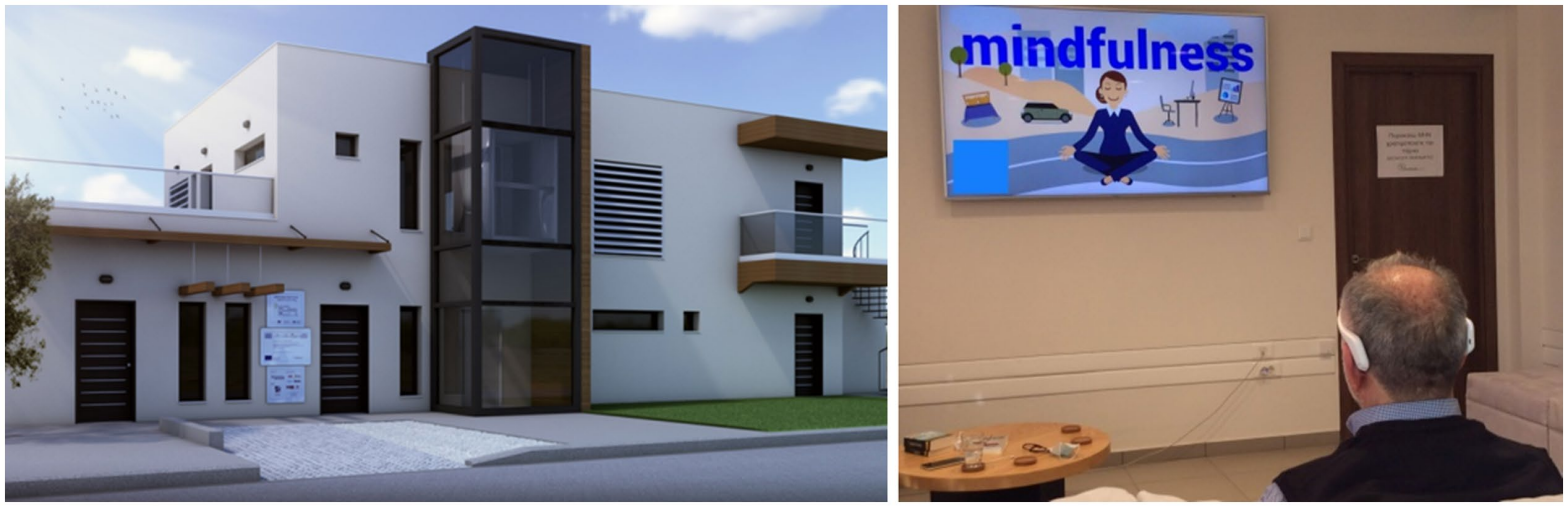
Smart home premises (left) and participant during the session (right).

For the purposes of the present study, we used the CERTH/ITI nZEB smart home^4^ premises. The smart home is a prototype and novel technologies demonstration infrastructure resembling a real domestic building where occupants can experience actual living scenarios while exploring various innovating smart IoT-based technologies with provided Energy, Health, Big Data, Robotics and Artificial Intelligence (AI) services (Figure 2). In the present study, we used the smart home environment in order to investigate the medication exercises remotely in a real home simulated environment. All participants visited the smart home only on weekdays so as to be able for the researchers to monitor and visit them in case of emergency. A psychologist-clinical research associate at CERTH visited the participants on a daily basis. The meditation sessions took place in the living room area where the participants could sit in a comfortable position on the couch while the meditations instructions were broadcasted on a TV set.

### 2.4. Data acquisition and processing

**Figure 3:**
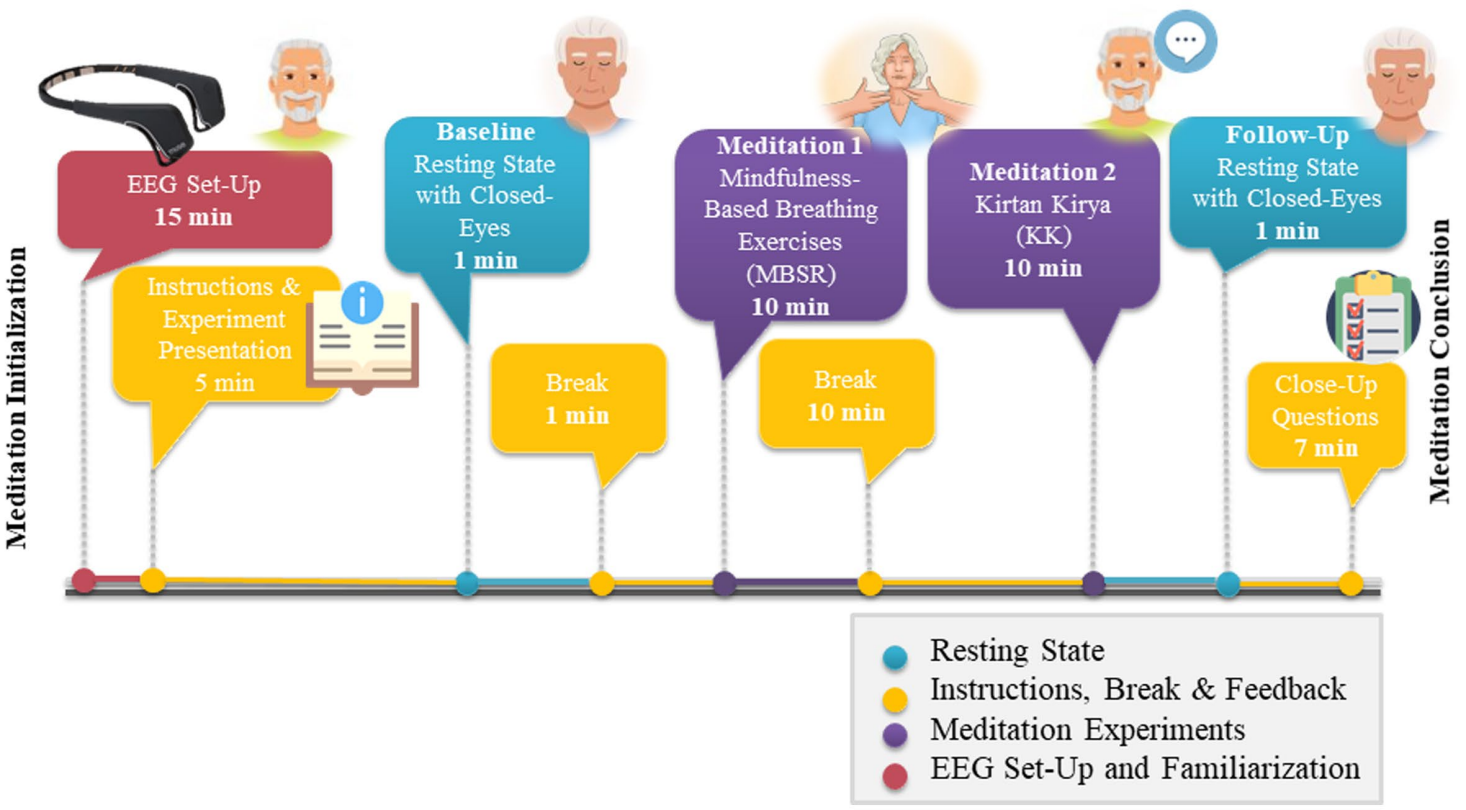
Electroencephalography (EEG) set-up and meditations process timeline.

The following four tasks were used to monitor participants’ brain activation, namely Session 1-RS Baseline, Session 2-MBSR, Session 3-KK and Session 4-RS Follow-Up. The whole duration of the experiment lasted approximately 50 min. Figure 3 illustrates the complete experimental procedure regarding the meditation session. Between MBSR and KK, a 10 min break where we explained the next procedure to the participant took place in order to avoid any counteract effect. During the preparation process before each session, a clinical research assistant was present at all times to position the Muse device properly on the participant and guide them through the process, at the same time ensuring they are comfortable and calm. A second research assistant was close by in order to monitor—in real time—the data collection of brain signals *via* a signal streaming visualization. Muse Lab Streaming Layer (LSL)^5^ was used for this purpose. Muse LSL is a Python package for streaming, visualizing, and recording EEG data from the Muse devices, developed by InteraXon. The research assistant monitored signals both during the placement of the device as well as during data collection. When artifacts due to various reasons (e.g., eye blinks, head movement) or misplacement of electrodes were present, the clinical researcher repeated the preparation process.

#### 2.4.1. Session 1 and session 4 resting state with eyes closed

One minute resting EEG activity (Baseline) was recorded for all the participants prior to the two meditation tasks and right after (Follow-up). During the resting-state EEG recording, participants were advised to keep themselves relaxed as much as possible, breathe normally, keep their eyes closed, sit still and minimize mouth movements and let their mind wander. The experimental procedure was monitored by a research assistant aiming to identify cases of horizontal eye movements, continuing blinking or excessive movement by visually inspecting the EEG traces during the experiment.

#### 2.4.2. Session 2: mindfulness-based stress reduction (MBSR)—witness the breath

In this meditation exercise, the participants were instructed to focus on their breath as it naturally and spontaneously moved through their body without consciously inhaling or exhaling. The practitioner of meditation progressively shifts their concentration from their chest motions to their nose, nostrils, and then to a tiny location just below their nostrils where the out-breath brushes their upper lip as it leaves. After focusing on the breath as an anchor, the meditator then draws attention to the breath’s different qualities, such as its temperature differences during the inhalation and exhalation as well as variations in its length and rhythm. When unimportant thoughts enter the mind during meditation, the practitioner acknowledges them and gently refocuses their attention elsewhere. We followed the procedures as suggested in (Lee et al., 2019; Dwivedi et al., 2021; Poos et al., 2022). More information about the MBSR breathing exercises that were applied can be found in the following link https://www.mindfulnessgreece.gr/.

#### 2.4.3. Session 3: Kirtan Kriya (ΚΚ) meditation

According to the original protocol (Khalsa and Newberg, 2021), The participants take a comfortable seat on a sofa, their feet resting on the ground. The goal of good posture is to be at ease when sitting with a straight spine that only curves naturally. KK employs “mantras,” or “tools for the mind,” which are noises that change in frequency or tone. Among the countless mantras, KK uses the five fundamental sounds: Saa, Taa, Na, and Maa, which the participants chant while breathing properly and maintaining a relaxed jaw. The participant listened to the 12-min prerecorded tape and proceeded in the following order: (a) sings aloud for 2 minutes; (b) sings in a stage whisper for 2 minutes; (c) talks to oneself in silence for 4 minutes; (d) whispers the sound for 2 minutes; and finally (e) sings the sound—for the last two minutes.

An adaptation of KK, i.e., the “Greek Kirtan Kriya” meditation, was created to appropriate this task to the local, Greek culture and language for the first time and for the purposes of this study, following the pace and instructions of the original. The original words were entirely foreign and unknown as regards to their (potential) meaning. Consequently, they caused discomfort to the participants and could alienate them to the task and the study. Therefore, we adapted the words to “La, Sol, Fa, Sol” i.e., the respective music notes, A, G, F, G, as spoken and taught in Greek music education, which felt familiar and meaning-neutral and -free to the participants, while also helping them with singing in-tune. The novel Greek adaptation of KK recording comprises of a sound track that features a professional singer, performing the sounds, and a video track that includes a calming image, the notes to sing along to and instructions to alternate the singing when needed. The video is available online^7^.

The EEG data were recorded using the MUSE 2 device (InterAxon Inc., Toronto, ON, Canada). The dry electrodes were placed according to the international 10–20 system in TP9, AF7, AF8, TP10 and FPz (as reference) and the sampling frequency was set to 220 Hz. Furthermore, this device is equipped with a 3-axis accelerometer for monitoring head movement. The device is, also, equipped with a rechargeable lithium-ion battery and its communication with other devices is performed through Bluetooth. In order to facilitate data collection and minimize errors Muse LSL was used, a Python package for streaming, visualizing, and recording EEG data from Muse devices developed by InteraXon (Alexandre et al., 2019). The data was captured in real-time and a researcher was stand-by to manually start the streaming of EEG data and observes the signal from each electrode. In this manner, the researcher was able to detect artifacts in the signal (e.g., rapid eye movement, tense facial expressions, etc.) early on and advise the participant to relax before each session starts.

The EEG analysis was conducted using MATLAB toolboxes. Noise and artifacts from raw EEG recordings were, respectively, removed by an infinite impulse response (IIR) linear phase 3–30 Hz bandpass filter and FORCe MATLAB toolbox, which removed artifact components from 1 s windows of the EEG signals *via* the following steps: (1) wavelet decomposition, (2) independent component analysis, and (3) thresholding. The Power Spectral Density (PSD) of the EEG was extracted by computing the Welch’s averaged modified periodogram. A sliding Hanning window of 1 s, with an overlap of 0.5 s, is applied to improve the estimation quality. The number of FFT points is set to 220 resulting in PSD estimates with frequency resolution of 1 Hz (the frequency sampling is 220 Hz). The resulting PSD for the electrode *i*, with *i* = 1,…, Nch, is a feature vector of NFT = 110 elements characterizing the power of the EEG oscillations from 0 up to 110 Hz. At this stage of our research we have not directly performed any kind of data normalization. However, indirectly, due to intensive data pre-processing analysis, averaging procedures have been used (i.e., averaged periodogram) or mean removal before the filtering. Furthermore, it is important to underline that the power of brain waves (alpha, beta, etc.) was calculated as the mean values over a range of frequencies (for example, the power of alpha waves is calculated as the mean value of frequencies from 8 to 13 Hz).

### 2.5. Statistical analysis

We compared the power of the brain waves (alpha, beta, and theta) from four different electrodes (TP9, TP10, AF7 and AF8) among the three groups at the level of significance *p* = 0.05. Statistical analysis was performed using SPSS v25.0 for Windows (IBM Corporation, Armonk, NY, United States). The results were compared between groups using Kruskal-Wallis analysis. For assessing the normality assumption for continuous and categorical variables we used the Kolmogorov–Smirnov and Chi-squared test, respectively. For examining the potential statistical significance between two independent groups (e.g., HC versus SCD), we used the Mann–Whitney test. We used Kruskal–Wallis in order to analyze the difference in the brain activity across the four groups for all sessions (Session 1-RS Baseline, Session 2-MBSR, Session 3-KK and Session 4-RS Follow-Up). In cases, where brain waves in particular electrodes showed statistical significance between sessions in each of the groups, we used Wilcoxon Signed rank test. Post-hoc Bonferroni corrections for the comparison of Different Meditation states within Group was used for multiple comparisons (α^critical value^ = α^family wise (0.05)^/c^number of comparisons (3)^ = 0.017) in order to avoid any false-positive result in the analysis. Correlation between neuropsychological tests and brain waves was assessed by using Pearson’s Correlation test. All descriptive statistics are reported with the mean and the standard deviation values (Table 3).

**Table 3:** Mean and standard deviation of the four meditation sessions of brain waves (theta, alpha, beta) across the three groups (HC = 13, SCD = 14, MCI = 13).

Diagnosis			HC		SCD		MCI		
Sessions	Frequency	Electrode	*M*	SD	*M*	SD	*M*	SD	*p*-value
Session 1—Resting State Baseline	Theta (Θ)	TP9	1.144	1.547	1.137	0.691	1.430	0.601	0.053
		AF7	0.946	0.411	1.299	0.641	1.071	0.499	0.289
		AF8	1.182	0.756	1.374	0.745	1.123	0.528	0.598
		TP10	0.881	0.502	1.273	0.534	1.438	0.899	**0.027**
	Alpha (a)	TP9	0.548	0.229	0.500	0.218	0.587	0.262	0.829
		AF7	0.474	0.152	0,460	0,152	0.464	0.171	0.297
		AF8	0.583	0.338	0.569	0.125	0.471	0.181	0.320
		TP10	0.608	0.221	0.615	0.169	0.603	0.241	0.952
	Beta (β)	TP9	0.244	0.079	0.220	0.095	0.255	0.065	0.714
		AF7	0.242	0.127	0,302	0,159	0.242	0.078	0.257
		AF8	0.302	0.147	0.335	0.151	0.297	0.098	0.807
		TP10	0.241	0.095	0.306	0.172	0.251	0.066	0.530
Session 2—MBSR	Theta (Θ)	TP9	0.947	0.628	1.135	0.488	1.385	0.791	0.262
		AF7	0.937	0.504	0.910	0.370	1.040	0.593	0.906
		AF8	0.884	0.284	1.222	0.624	1.073	0.759	0.436
		TP10	0.843	0.483	1.049	0.440	1.537	1.231	**0.007**
	Alpha (a)	TP9	0.606	0.190	0.565	0.202	0.617	0.256	0.905
		AF7	0.531	0.189	0.480	0.166	0.462	0.204	0.467
		AF8	0.568	0.340	0.534	0.175	0.492	0.214	0.859
		TP10	0.673	0.250	0.642	0.181	0.762	0.285	0.311
	Beta (β)	TP9	0.222	0.068	0.204	0.058	0.242	0.070	0.585
		AF7	0.196	0.081	0.228	0.086	0.247	0.110	0.405
		AF8	0.261	0.126	0.241	0.105	0.282	0.135	0.756
		TP10	0.215	0.076	0.218	0.066	0.268	0.082	0.206
Session 3—KK	Theta (Θ)	TP9	1.460	0.564	1.489	0.584	1.513	0.801	0.988
		AF7	1.319	0.479	1.347	0.492	1.312	0.756	0.787
		AF8	1.517	0.620	1.572	0.799	1.470	0.615	0.961
		TP10	1.484	0.457	1.558	0.551	1.475	0.567	0.966
	Alpha (a)	TP9	0.631	0.197	0.604	0.136	0.674	0.444	0.657
		AF7	0.566	0.174	0.533	0.071	0.514	0.303	0.213
		AF8	0.688	0.262	0.575	0.120	0.595	0.259	0.502
		TP10	0.677	0.198	0.661	0.153	0.607	0.229	0.893
	Beta (β)	TP9	0.317	0.103	0.266	0.066	0.332	0.132	0.193
		AF7	0.381	0.141	0.312	0.112	0.340	0.145	0.346
		AF8	0.499	0.206	0.343	0.119	0.439	0.183	0.055
		TP10	0.355	0.108	0.308	0.110	0.320	0.110	0.250
Session 4—Resting State Follow-Up	Theta (Θ)	TP9	1.288	2.066	1.307	0.665	1.251	1.576	0.080
		AF7	1.391	2.007	0.977	0.431	1.025	0.618	0.893
		AF8	0.889	0.601	1.250	0.701	1.204	1.251	0.226
		TP10	1.262	1.571	1.479	1.202	1.519	1.636	0.426
	Alpha (a)	TP9	0.761	1.106	0.595	0,132	0.560	0.246	0.174
		AF7	0.722	1.035	0.562	0.229	0.424	0.176	0.691
		AF8	0.510	0.240	0.510	0.106	0.468	0.180	0.589
		TP10	0.771	0.772	0.664	0.331	0.639	0.303	0.710
	Beta (β)	TP9	0.296	0.187	0.302	0.113	0.243	0.072	0.433
		AF7	0.332	0.203	0.360	0.267	0.242	0.074	0.058
		AF8	0.348	0.175	0.311	0.137	0.301	0.119	0.806
		TP10	0.342	0.179	0.336	0.133	0.261	0.075	0.431

## 3. Results

### 3.1. Comparison between groups

**Figure 4:**
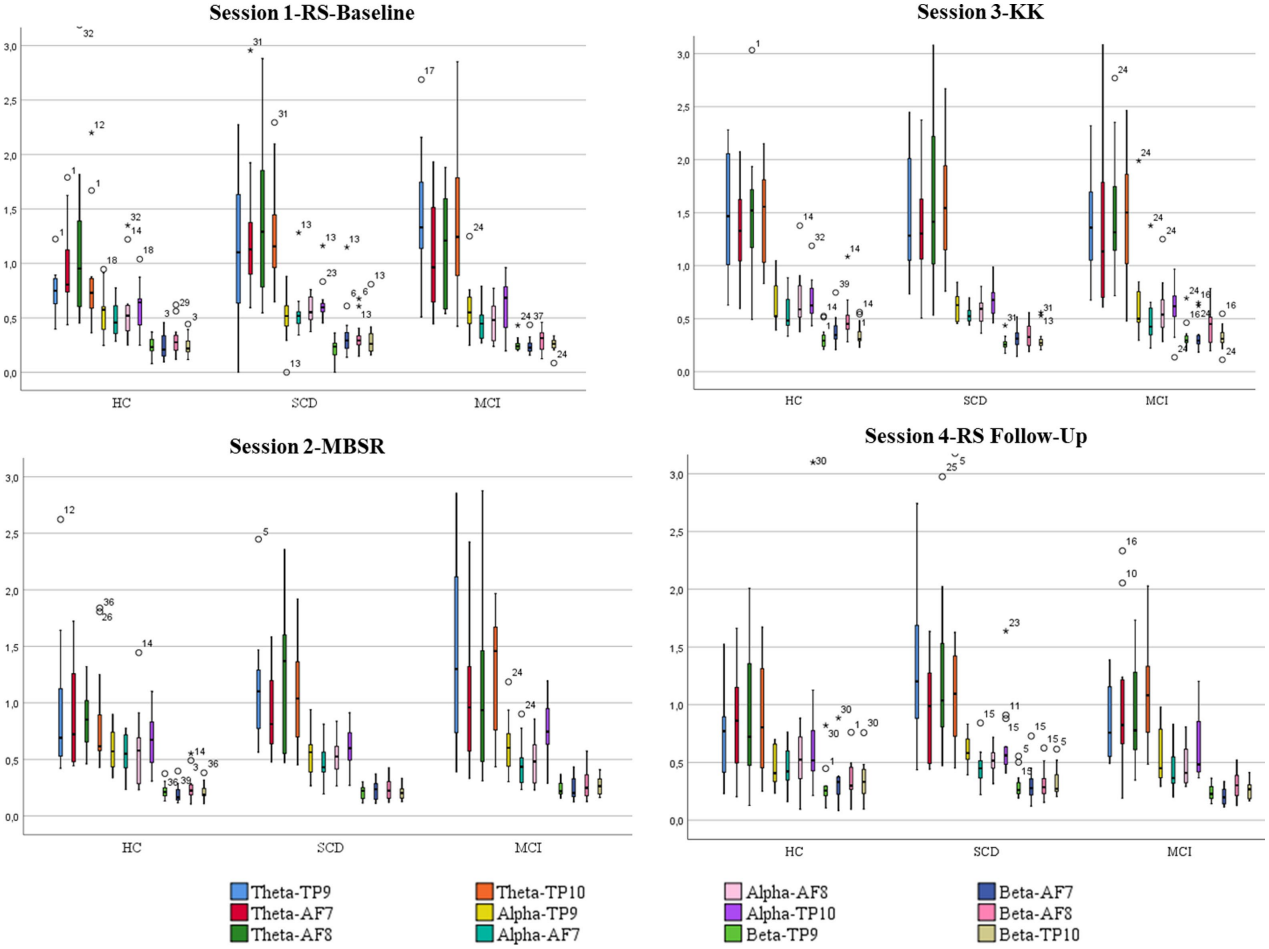
Boxplots showing mean values and standard deviations for each one of the four 411 sessions and power of brain waves (alpha, theta, and beta) of the AF7, AF8, TP9 and TP10 412 electrodes among HC, SCD and MCI groups.

Table 3 and Figure 4 present the results after comparing the three different groups for the three different meditation tasks. In particular, the results of the Kruskal-Wallis test showed that there was statistically significant difference across the three groups of participants at level *p* = 0.050, such as: (i) in Session 1-RS Baseline in theta frequency at TP9 and TP10 H (2) = 5.892, *p* = 0.050 and H (2) = 7.254, *p* = 0.027, (ii) in Session 2—MBSR in theta waves TP10 H (2) = 5.261, *p* = 0.007, (iii) trend in Session 3-KK in beta frequency AF8 H (2) = 4.715, *p* = 0.055, and (iv) in Session 4-RS Follow-Up in theta waves TP9 and trend in Beta AF7 H (2) = 5.044, p = 0.050 and H (2) = 4.866, *p* = 0.058, respectively. In addition, the results of the Mann–Whitney indicate that statistically significant differences are also present between pairs of groups. In particular, according to Post-hoc Mann–Whitney test, theta waves in Session 1 at TP9 and TP10 were higher for SCD compared to HC (*U* = 39.00, *p* = 0.034) and (*U* = 38.00, *p* = 0.03) respectively, whereas beta waves at AF8 in Session 3-KK were higher for HC compared to SCD (*U* = 41.00, *p* = 0.026). Similarly, theta waves in Session 1 at TP9 and TP10 were higher for MCI compared to HC (*U* = 37.00, *p* = 0.015 and *U* = 37.00, *p* = 0.015), as well as in Session 2-MBSR theta waves at TP10 were higher for MCI compared to HC (*U* = 43.00, *p* = 0.057). On the other hand, beta waves in Session 4-RS Follow-Up were higher for HC compared to MCI (*U* = 38.00, *p* = 0.052; Figure 4).

### 3.2. Comparison of different meditation states within group

*Session 1—Resting State Baseline vs. Session 2—MBSR:* No statistical differences were found in HC between Session 1-RS baseline and Session 2—MBSR. However, there was a trend of reduced Βeta waves in Session 2-MBSR compared to Session 1-RS baseline at TP10 *Z* = –1.647, *p* = 0.059. For SCD, Wilcoxon Signed-ranks test indicated that Theta and Beta waves at TP10 in Session 2-MBSR was statistically significantly reduced compared to Session 1-RS Baseline *Z* = –2.201, *p* = 0.028 and *Z* = −2.411, *p* = 0.016, respectively. Moreover, Beta waves at AF8 in Session 2-MBSR was statistically significantly reduced compared to Session 1-RS *Z* = –2.481, *p* = 0.013. For MCI, Wilcoxon Signed-ranks test indicated that Alpha waves at TP10 in Session 2-MBSR was statistically significantly higher than Session 1-RS Z = -2.041, *p* = 0.041.

*Session 1—Resting State Baseline vs. Session 3-KK:* On the other hand, Wilcoxon Signed-ranks test indicated for HC that Theta waves at TP9, TP10 and AF7, AF8 in Session 3-KK was statistically significantly reduced compared to Session 1-RS *Z* = –2.271, *p* = 0.023, *Z* = −3.110, *p* = 0.002 and Z = −2.341, *p* = 0.019, Z = −2.132, *p* = 0.033, respectively. Moreover, Alpha waves at TP9, AF7 and AF8 in Session 3-KK was statistically significantly higher than Session 1-RS Z = -1.852, *p* = 0.064, Z = -2.411, *p* = 0.016 and Z = -2.062, *p* = 0.039, respectively. Finally, with respect to Beta waves at TP9, TP10 and AF7 and AF8 statistically significantly higher values were found in Session 3-KK compared to Session 1-RS Z = -1.852, p = 0.064, Z = -2.621, *p* = 0.009 and Z = -2.551, *p* = 0.011, Z = -2.551, p = 0.011, respectively. Similarly for SCD, Theta waves at TP9 and TP10 in Session 3-KK was statistically significantly reduced compared to Session 1-RS Z = -2.201, p = 0.028 and Z = −2.062, p = 0.039, respectively. Also, Alpha waves at TP9 in Session 3-KK showed a trend of statistically significantly higher values than Session 1-RS Z = -1.922, *p* = 0.055. Finally for MCI Theta waves at AF8 in Session 3-KK was statistically significantly reduced compared to Session 1-RS Z = -1.961, *p* = 0.050. Also, Beta waves at TP9, TP10 and AF7 and AF8 in Session 3-KK was statistically significantly higher than Session 1-RS Z = -2.51, *p* = 0.012, Z = -2.43, *p* = 0.015 and Z = -2.27, p = 0.023 and Z = -2.90, *p* = 0.004, respectively.

*Session 1—RS Baseline vs. Session 4—RS Follow-Up:* Statistical analysis indicated that Beta waves at TP10 in Session 4-RS Follow-up was statistically significantly higher than Session 1-RS Baseline Z = -2.197, *p* = 0.028 for HC. For SCD, statistical analysis indicated that Alpha waves at AF7 in Session 4-RS Follow-up was statistically significantly higher than Session 1-RS Baseline Z = -2.667, *p* = 0.008. Regarding MCI, no statistical differences were found between the Session 1-RS Session 4-RS Follow-Up for MCI.

*Session 2—MBSR vs. Session 3—KK:* For HC, statistical analysis showed that Theta waves at TP9, TP10 and AF7, AF8 in Session 3-KK was statistically significantly higher compared to Session 2-MBSR Z = -2.481, p = 0.013, Z = −2.970, *p* = 0.003 and Z = −2.900, p = 0.004, Z = −3.040, *p* = 0.002, respectively. Moreover, Alpha waves at AF8 in Session 3-KK was statistically significantly higher than Session 2-MBSR Z = -2.132, *p* = 0.033. Finally, with respect to Beta waves at TP9, TP10 and AF7 and AF8 statistically significantly higher values were found in Session 3-KK compared to Session 2-MBSR Z = -2.411, p = 0.016, Z = -2.970, p = 0.003 and Z = -3.180, *p* = 0.001, Z = -3.110, p = 0.002, respectively. Moreover, for SCD, statistical analysis indicated that Theta waves at TP9, TP10 and AF7, AF8 in Session 3-KK was statistically significantly higher compared to Session 2-MBSR Z = -2.341, *p* = 0.019, Z = −2.830, *p* = 0.005 and Z = −2.830, p = 0.005, Z = −2.132, p = 0.033, respectively. Moreover, with respect to Beta waves at TP9, TP10 and AF7 and AF8 statistically significantly higher values were found in Session 3-KK compared to Session 2-MBSR Z = -2.970, p = 0.003, Z = -2.970, p = 0.003 and Z = -2.830, p = 0.005, Z = -3.110, p = 0.002, respectively. Finally for MCI, statistical analysis indicated that Theta waves at AF8 in Session 3-KK was statistically significantly higher compared to Session 2-MBSR Z = -2.275, *p* = 0.023. Moreover, with respect to Beta waves at TP9, AF7 and AF8 statistically significantly higher values were found in Session 3-KK compared to Session 2-MBSR Z = -2.824, p = 0.005, Z = -2.197, p = 0.028 and Z = -3.059, p = 0.002, respectively.

*Session 2—MBSR vs. Session 4—RS Follow-Up:* Statistical analysis indicated that Beta waves at AF7 and TP10 in Session 4-RS Follow-up was statistically significantly higher than Session 2-MBSR Z = -2.824, p = 0.005 and Z = -2.589, *p* = 0.010, respectively, for HC. For SCD, statistical analysis indicated that Beta waves at TP9 and TP10 in Session 4-RS Follow-up was statistically significantly higher than Session 2-MBSR Z = -2.432, *p* = 0.015 and Z = -2.589, p = 0.010, respectively.

*Session 3—KK vs. Session 4—RS Follow-Up:* Finally, regarding Session 3-KK and Session 4-RS Follow-up HC, showed lower values in Theta waves at TP9 in Session 4 Z = –1.961, *p* = 0.050. For SCD, statistical analysis indicated that Theta and Alpha waves at TP9 in Session 4-RS Follow-up was statistically significantly lower than Session 3-KK Z = –2.040, *p* = 0.041 and Z = –2.824, p = 0.005, respectively. For MCI Statistical analysis indicated that Beta waves at AF7 in Session 4-RS Follow-up was statistically significantly lower than Session 3-KK Z = –2.312, *p* = 0.021.

**Figure 5:**
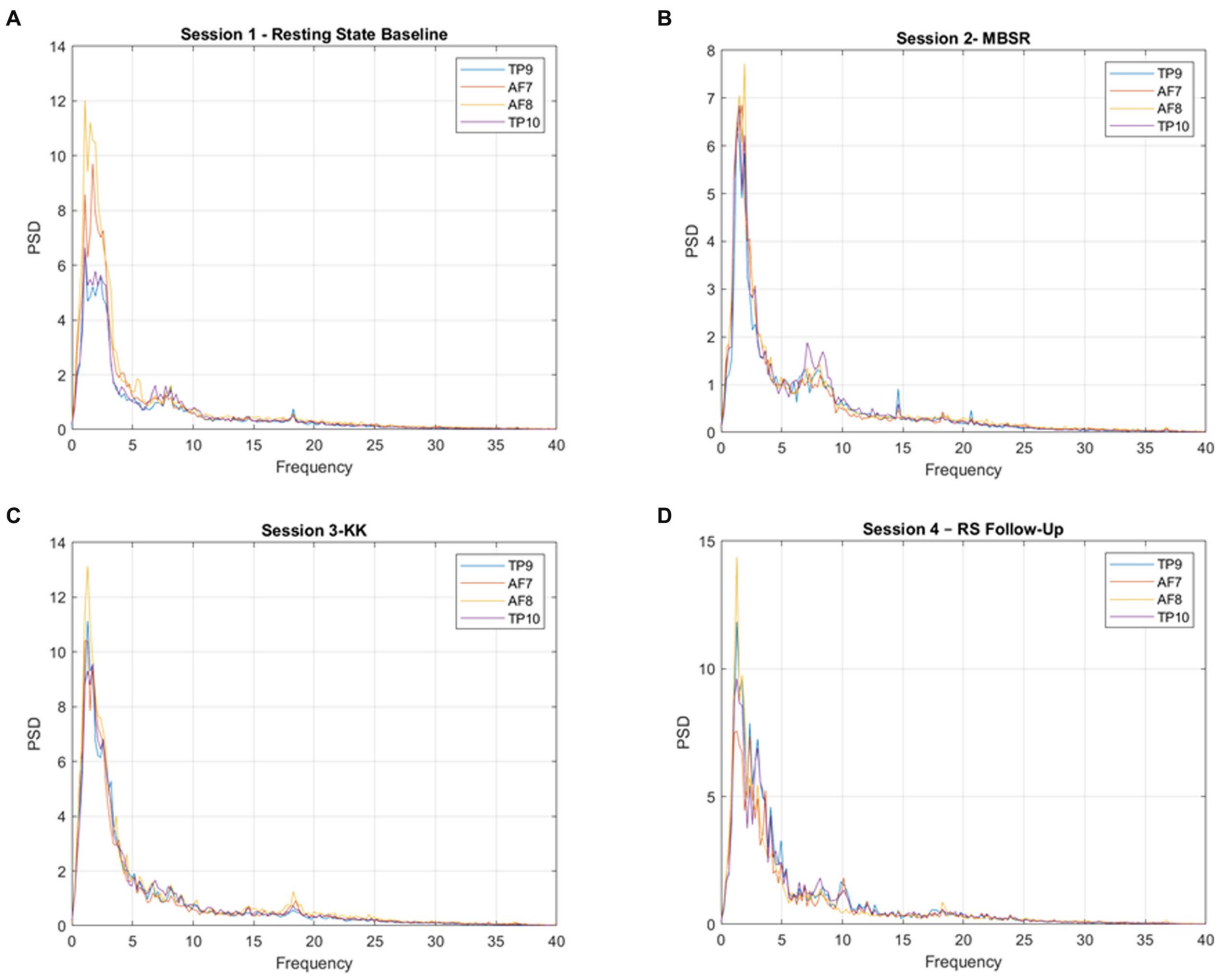
Power spectral density (PSD) for each channel, averaged across participants, for **(A)** Session 1-RS Baseline, **(B)** 480 Session 2-MBSR, **(C)** Session 3-KK, and **(D)** Session 4-RS Follow-Up.

Figure 5 presents the waveforms during the meditation sessions (Session 1-RS Baseline, Session 2-MBSR, Session 3-KK and Session 4-RS Follow-Up) from each channel (AF7, AF8, TP9, TP10). As a result, HC present higher activation during the tasks while SCD and MCI present a similar activation pattern.

**Figure 6:**
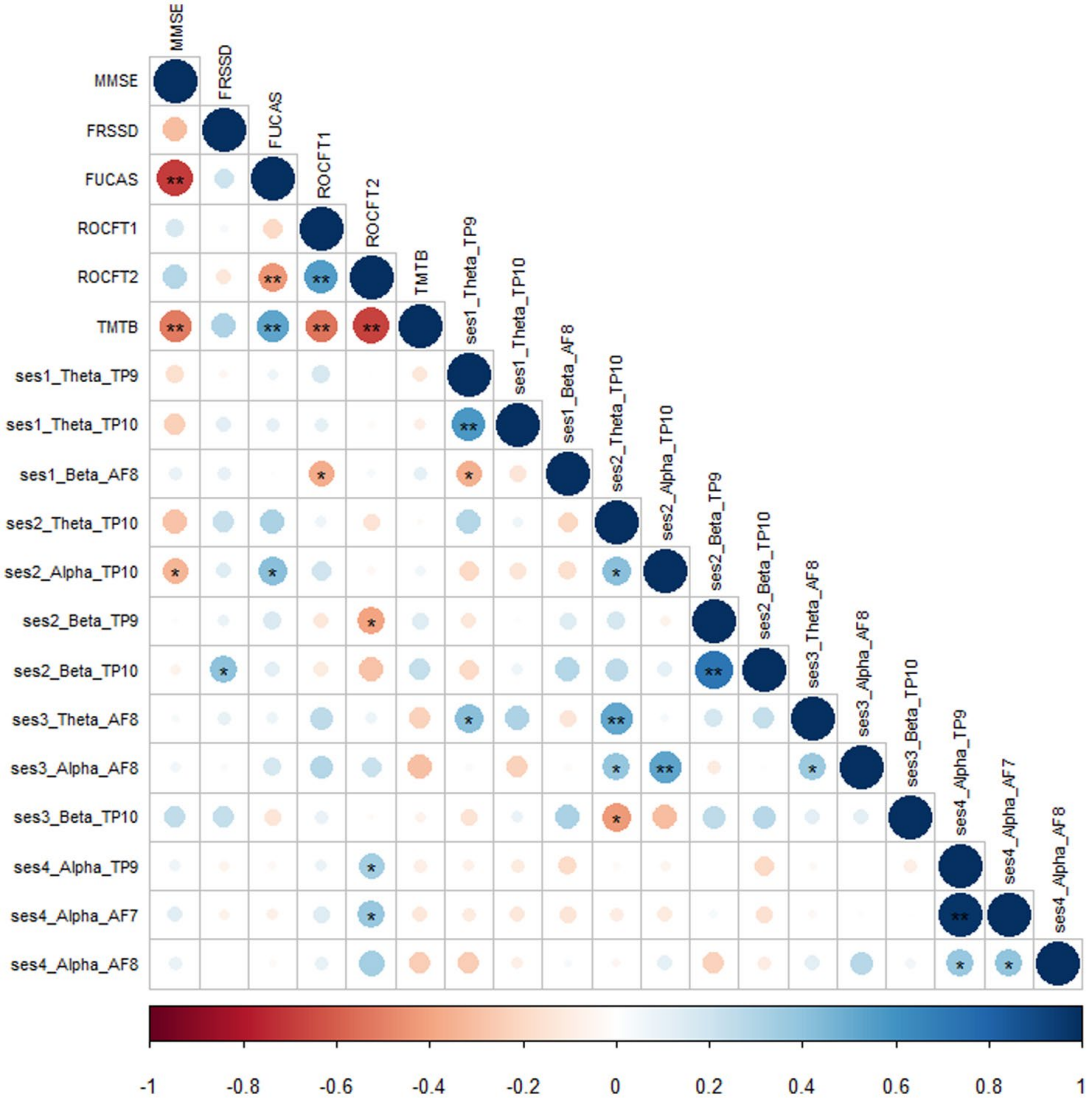
Correlation matrix between sessions, brain waves, electrodes and neuropsychological 490 tests.

Moreover, we calculated absolute significant correlations ranged from − 1 to 1 after applying Pearson correlation, representing small to medium strength relationships between the four meditation sessions, brain frequencies and neuropsychological tests (Figure 6). In particular, we found strong correlation between cognitive tests assessing general cognitive function. Visuospatial ability and visuo-construction statistically significantly correlated with theta and alpha waves at Session 1—RS Baseline and Session 2-MBSR, respectively, at TP10, while beta and theta waves in KK at AF8 were also positively correlated with neuropsychological tests.

## 4. Discussion

The present study highlights the results of investigating two meditation techniques in the preclinical stages of AD ranging from SCD to MCI compared to HC by using a portable EEG (Muse) for the first time in a smart-home environment. Our results showed that brain waves during meditation sessions (MBSR and KK) were significantly associated in between groups mostly in theta and beta waves in a similar fashion for HC as well as in SCD and MCI. Moreover, in our study we provide evidence that portable EEG has the capabilities to be potentially used as part of a classification through meditation, given that we were able to perceive altered activation across different meditation tasks between HC, SCD and MCI.

In summary, our results demonstrate that the Muse EEG can be used to quickly obtain EEG data from a number of people providing a viable methodology for potential clinical applications such as brain activation assessment of early cognitive decline related to AD (van Lutterveld et al., 2017). The connections between EEG and cognitive stage that we discovered are consistent with previous studies. For instance, we discovered that compared to HC, SCD and MCI showed greater theta oscillations over the temporo-parietal regions during the RS baseline and MBSR session. Even though the KK session was a more active meditation task, HC and SCD participants showed higher beta frequencies over frontal areas than the MCI participants, who had more severe cognitive impairment. In addition, we found that in HC and SCD compared to MCI, follow-up RS sessions showed higher theta oscillations across temporo-parietal and increased beta oscillations over frontal areas. After conducting a within-group analysis, we discovered a parallel association that supports this relationship. For instance, we discovered that theta and beta frequencies among SCD decreased throughout MBSR and KK sessions compared to RS-Baseline, as opposed in HC compared to SCD and MCI, we observed an increase in alpha and beta frequencies during meditation sessions (MBSR and KK) which was less pronounced compared to RS-baseline. We also found the similar association in KK only among subjects where an increase in theta and beta was shown in both SCD and MCI but not in alpha frequency as in HC, which is, to the best of our knowledge, a novel observation.

The relationships we observed between cognitive stages and EEG power for the most part were in line with previous findings. Specifically, in line with findings from other related approaches, we observed an increase in frontal theta EEG power with increased cognitive decline (Aftanas and Golocheikine, 2002; Babiloni et al., 2013; Dissanayaka et al., 2015; Sharma et al., 2019). The posterior alpha power increased in SCD and HC but not in MCI, as well. The majority of previous research approaches investigated EEG power during RS, but in this study, we examined EEG power during actual MBSR and KK performance as well as regular breathing in RS at baseline and follow-up to investigate the effects of meditation. Furthermore, it is generally known that alpha power decreases while performing a task, most likely because of an increased focus on visuospatial attention (Sauseng et al., 2005; Klimesch, 2012). With this in mind, we suggest that the reduction in alpha power that we observed with increased cognitive decline reflects a greater demand on attentional resources to achieve successful performance when one has early cognitive impairment. Our results involving EEG are relatively consistent with other related studies (Pagnoni et al., 2008; Braboszcz and Delorme, 2011; van Lutterveld et al., 2017; Sharma et al., 2019). Therefore our results suggest that a shift in attentional focus that occurs during a mindfulness practice (e.g., redirecting attention one’s current breathing) may underlie the inability to sustain attention, which is a condition affected in early stages of cognitive impairment across AD (Lazarou et al. 2019b, 2020). Our findings are in line with previous established evidence that structural changes in regions of the brain may affect brain electrical activity related to cognitive impairment in older adults.

Additionally, the EEG study showed that during the KK meditation session of SCD and MCI compared to HC, there was a widespread increase in beta, theta, and in some cases, alpha EEG power. In the right posterior regions, the power of the alpha EEG increased during MBSR and KK in comparison to HC. However, compared to HC, beta was lower in the meditation groups for cognitively impaired people. The EEG data supports the already existing evidence that alpha and theta EEG power are increased during the meditative state. The alpha wave is associated with relaxation and memory consolidation (Aftanas and Golocheikine, 2002; Nunez and Srinivasan, 2009; Ahani et al., 2014; Xue et al., 2014; Kaur and Singh, 2015; Innes et al., 2018; Sharma et al., 2019) and the theta band is related with focused attention, cognitive processing, creativity, and memory, but also with drowsiness (Moretti et al., 2004; Baijal and Srinivasan, 2010; Stapleton et al., 2020). Therefore, it seems that meditation weakens the regions in charge of top-down regulation while strengthening the ones in charge of stimulus-driven reactions. The few studies that have examined the cognitive benefits of meditation in MCI or AD patients have yielded mixed results, with some demonstrating no appreciable improvement over HC (Klainin-Yobas et al., 2019; Larouche et al., 2019). It is possible that a longer follow-up would bring out the cognitive benefits, as has been noted in some of the studies (Quintana-Hernández et al., 2016; Wong et al., 2017). On the other hand a recent study in healthy young participants, showed that intervention Mindfulness groups demonstrated significant improvement in mental health, while there was no correlation between mindfulness score and either EEG measure, at either baseline or over time (Acabchuk et al., 2021). Thus, it can be hypothesized that cognitively demanding tasks like KK, which require the storage and processing of information for longer period of time than RS, may need a more persistent coupling between alpha and theta rhythms.

The present findings unequivocally show the viability and potential of this strategy, although more research will be required to advance our understanding of EEG markers of meditation. The portable EEG and meditation sessions have some preliminary support from these results, but further research is required to confirm and build on these outcomes. Additionally, a bigger sample size would be optimal in order to guarantee the reproducibility and generalizability of our findings. However, given the novelty of the study both regarding the set-up and design as well as the participants’ diagnosis (few reported studies in SCD using portable EEG), we believe that the results are clinically meaningful and highlight the importance of the neurophysiological studies in AD research. Another limitation is that the portable EEG’s assessment was restricted to certain brain regions due to the number of available electrodes. Given the variety of techniques used in neurophysiological research, it may be challenging to compare our findings to those of other similar methodologies in this setting. The EEG provides a large deal of experimental versatility in terms of processing, as has recently been reviewed, but the wide range of parameters indicates that results are frequently incomparable. There is lack of standardization of signal processing techniques and the meditation practices assessed with EEG. Lomas et al. (2015) stated in a systematic review of mindfulness meditation using EEG that one of the issues was the great diversity of methods, given that no more than three studies employed the same processing. These inconsistencies in the literature hinder the formulation of more specific hypotheses and restrict studies exploring complementary features of the EEG signal to an observational level. Despite the abovementioned limitations, implementation of Muse was acceptable and feasible to be used by 40 people with SCD, MCI as well as HC during their visit in a smart-home environment. Nonetheless, the results of this study may be considered to provide meaningful evidence suggesting that meditation based interventions represent a viable, flexible, accessible option for individuals experiencing cognitive impairment, as indicated by EEG derived biomarkers.

## 5. Conclusion

To best of our knowledge this is the first reported study to investigate brain response of HC, SCD and MCI, in a variety of meditation sessions, including the adapted Greek Kirtan Kriya meditation introduced in this study, to explore changes among different groups. Moreover, this is the first study to measure them in a smart home setting/portable (as opposed to lab setting/lab EEG) and using a novel meditation protocol translated in Greek language. Overall results suggested the meditation intervention had large varying effects on EEG brain waves, and the speed of change from pre-meditation to post-meditation states of the EEG was significant across early cognitive decline participants. However, results from such a small group of participants should be interpreted with caution and should be verified in future research with a larger sample size. Nevertheless, the cerebral activities of cognitive processing in people at the early stages of AD remain undetermined and more research is needed to verify these findings to further explore the use of EEG signals as a biomarker. Findings from this study reveal the potential application of a portable EEG device as a low cost, non-invasive and easily accessible clinical tool for people with cognitive impairment. As a future direction, researchers could re-use the protocol of meditation and portable or lab EEG to explore longitudinal effects of the interventions.

## Data availability statement

The datasets presented in this article are not readily available because the dataset is being processed currently till the end of the RADAR-AD project. Requests to access the datasets should be directed to iouliettalaz@iti.gr.

## Ethics statement

The studies involving human participants were reviewed and approved by Ethical committee of Centre for Research and Technology Hellas (CERTH; ETH.COM 54/17-06-2020) Scientific and Ethics Committee of Greek Association of Alzheimer’s Disease and Related Disorders (GAADRD; 242/2022 ΑΙ_07/10/2021). The patients/participants provided their written informed consent to participate in this study.

## Author contributions

SN, IK, IL, VO, TS, and LM contributed to the conception and design of the study. IL, VO, LM, MG, VA, and SN organized the whole set-up and experiment, constructed the dataset, and analyzed the results. MT examined the participants and set the diagnosis. IL, VO, and LM performed the statistical analysis. IL, VO, and SN wrote the first draft of the manuscript and wrote sections of the manuscript. IK, AB, and MT made the final corrections. The RADAR-AD Consortium approved the submission of the present manuscript. All authors contributed to the article and approved the submitted version.

## Funding

This research was funded by the RADAR-AD project, which has received funding from the Innovative Medicines Initiative 2 Joint Undertaking under grant agreement No. 806999. This Joint Undertaking receives support from the European Union’s Horizon 2020 research and innovation programme and EFPIA (https://www.imi.europa.eu). This communication reflects the views of the RADAR-AD Consortium and neither IMI nor the European Union and EFPIA are liable for any use that may be made of the information contained herein.

## Conflict of interest

The authors declare that the research was conducted in the absence of any commercial or financial relationships that could be construed as a potential conflict of interest.

## Publisher’s note

All claims expressed in this article are solely those of the authors and do not necessarily represent those of their affiliated organizations, or those of the publisher, the editors and the reviewers. Any product that may be evaluated in this article, or claim that may be made by its manufacturer, is not guaranteed or endorsed by the publisher.

## Footnotes

^1^ https://choosemuse.com/

^2^ https://www.radar-ad.org/

^3^ http://www.alzheimer-hellas.gr/index.php/el/

^4^ https://smarthome.iti.gr/

^5^ https://zenodo.org/record/3228861#.ZB2x63ZBy3A

^7^ https://www.youtube.com/watch?v=yql7Z3SKX-Y&feature=youtu.be&ab_channel=BuddhaLibreYogaStudio
